# Y-RNAs and their fragments reflect kidney injury in diabetic kidney disease

**DOI:** 10.1016/j.omtn.2026.102998

**Published:** 2026-07-01

**Authors:** Qiao Zhao, Yunyi Liang, Rudmer Postma, Jacques M.G.J. Duijs, Juliette A. de Klerk, Leen M. ’t Hart, Joris I. Rotmans, Anton Jan van Zonneveld, Roel Bijkerk

**Affiliations:** 1 Department of Internal Medicine (Nephrology) and the Einthoven Laboratory for Vascular and Regenerative Medicine, Leiden University Medical Center, Leiden, the Netherlands; 2 Nephrology Department, General Hospital of Northern Theater Command, Shenyang City, China; 3 Department of Cell and Chemical Biology, Leiden University Medical Center, Leiden, the Netherlands

**Keywords:** MT: non-coding RNAs, Y-RNA, diabetic kidney disease, vascular injury, extracellular vesicles, high-density lipoprotein, Argonaute 2, circulation, biomarker

## Abstract

Diabetic kidney disease (DKD) affects one-third of patients with diabetes mellitus (DM). Y-RNAs (RNYs) are a type of highly abundant non-coding RNA that could potentially serve as disease biomarkers. This study investigated whether circulating RNYs associate with DKD that may facilitate early detection and intervention of DKD. Patients were categorized into three groups: healthy controls (HCs), patients with type 1 DM without DKD, and those with DKD. We examined the levels of RNYs and their fragments using RT-qPCR. Levels of RNYs were determined in three main non-coding RNA carriers (extracellular vesicles [EVs], AGO2, and HDL). Serum expression of full-length RNYs mainly decreased in the DKD group, while RNY fragments were elevated in the DM group compared to the HC group and decreased in the DKD group compared to the DM group. While we observed circulating RNYs to be attached to AGO2 and HDL, the majority is present in EVs. In addition, the subtype ratio of full-length RNY3/RNY4 associated with kidney injury, while RNY1, RNY3, RNY3/RNY4 ratio and all fragment RNYs strongly associated with markers of vascular injury. Circulating RNYs and their fragments associate with (vascular injury in) DKD show potential as biomarkers for kidney injury in DM.

## Graphical abstract

**Figure:**
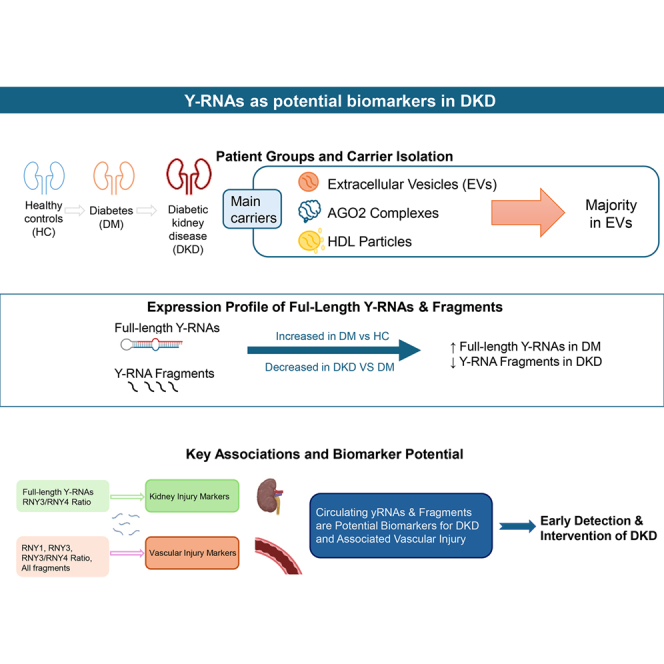

## Teaser

This study identifies circulating YRNA, and fragments derived thereof, as previously unrecognized potential biomarkers of diabetic kidney disease. Predominantly carried by extracellular vesicles, altered YRNA profiles and in particular the RNY3/RNY4 ratio is associated with kidney injury and vascular dysfunction, highlighting their diagnostic potential.

## Introduction

Diabetic kidney disease (DKD) is one of the most common microvascular complications associated with diabetes mellitus (DM).^1^ According to the World Health Organization (WHO), the incidence of DKD among patients with diabetes ranges from 30% to 40%, with this proportion on the rise.^2^ As the number of patients with diabetes increases, DKD has become a significant challenge in the field of global public health, with both its global prevalence and mortality rates showing an upward trend. DKD severely impairs kidney function and significantly affects patients' quality of life, being one of the leading causes of end-stage kidney disease (ESKD).^2^ The molecular mechanisms behind DKD are complex, involving chronic inflammation, oxidative stress, and fibrosis.^3^^,^^4^^,^^5^^,^^6^ Given the severity and complexity of DKD, the identification of effective biomarkers for early diagnosis and intervention is crucial. Ideal biomarkers should possess high sensitivity and specificity, capable of detecting kidney damage at the early stages of DKD, even before clinical symptoms emerge. Additionally, the identification and validation of therapeutic targets are equally important for the development of new treatment methods. These targets may involve multiple pathways, including inflammation, oxidative stress, and cell signaling.^5^^,^^7^ Such novel interventions may more effectively control the progression of DKD, reduce patient mortality, and improve quality of life, thereby timely intervening to delay disease progression and reduce the risk of complications for patients.^3^^,^^4^

Non-coding RNAs (ncRNAs) are a class of RNA molecules that do not encode proteins but play various biological regulatory functions through interactions with other nucleic acids or proteins. In recent years, typical ncRNAs such as microRNAs (miRNAs), lncRNAs, and circular RNAs (circRNAs) have received considerable attention,^8^ yet many “atypical” RNA molecules remain to be further investigated. Y-RNAs, also called RNYs, are such a class of non-coding small RNA molecules, approximately 83–112 nt in length, initially discovered in the serum of systemic lupus erythematosus (SLE) patients as components of the RNA in circulating ribonucleoprotein (RNP) autoantigens Ro60 and La.^9^^,^^10^^,^^11^ RNYs exhibit good sequence conservation and cross-species conservation.^12^ In the human genome, four RNY members have been identified: RNY1, RNY3, RNY4, and RNY5, while RNY2 has been excluded from the RNY family as it was proven to be a degradation fragment of RNY1. These RNY gene clusters are located on chromosome 7, with RNY1 on the negative strand and RNY3, RNY4, and RNY5 on the positive strand. RNYs fold into a hairpin-like secondary structure similar to pre-miRNA after transcription,^13^ while RNYs are also cleaved into smaller fragments, though their cleavage mechanisms may differ from that of pre-miRNA.^14^ Full-length RNYs (FL RNYs) and their derived small fragments bind to various carriers to exert functions within cells or are transported to the extracellular environment.^15^^,^^16^ Intracellularly, RNYs play key roles in multiple biological processes, including DNA replication, chromatin remodeling, and post-transcriptional modifications.^17^ In the extracellular environment, RNYs are highly abundant and can be detected in plasma and ncRNA carriers extracted from plasma, such as extracellular vesicles (EVs).^18^^,^^19^^,^^20^ In circulating samples from patients with cancer, cardiovascular diseases, and autoimmune diseases, specific RNYs and their derived fragments have been found to be dysregulated, suggesting their potential as diagnostic markers.^21^^,^^22^^,^^23^^,^^24^^,^^25^

Given this established role of RNYs and their presence in various ncRNA carriers, we were interested in exploring the potential of RNYs as biomarkers for DKD. Understanding the role of RNYs in DKD may reveal new avenues for early diagnosis, prognosis, and eventually therapeutic intervention to address this debilitating disease.

## Results

### Y-RNA can be fragmented

RNYs are ubiquitously present in the bloodstream in various forms and can be detected in the circulation using current sequencing technologies. However, the precise sequences and exact lengths of these fragments remain challenging to ascertain accurately.^16^ Additionally, the presence of numerous pseudogenes of RNYs in the genome contributes to the inaccuracy in the alignment of sequencing data with genomic locations.^26^ To gain a deeper understanding of RNYs within the body, we utilized small RNA sequencing (RNA-seq) data from human circulation and manually aligned it to the genomic loci of RNYs. Through this alignment, we identified not only FL RNYs but also fragment RNYs (f RNYs) derived from 5′ terminal regions and 3′ terminal regions (Figure S1A). Based on sequencing data, the 5′ terminal fragments were found to be the predominant form of f RNYs. As illustrated schematically (Figures S1B and S1C**)**, RNYs undergo intracellular processing into these multiple fragment subtypes, which are subsequently released into the extracellular circulation via diverse carriers.^10^^,^^14^^,^^20^^,^^27^^,^^28^^,^^29^^,^^30^

### Differential expression of FL RNYs as well as fragmented RNYs between DM and DKD

**Figure 1:**
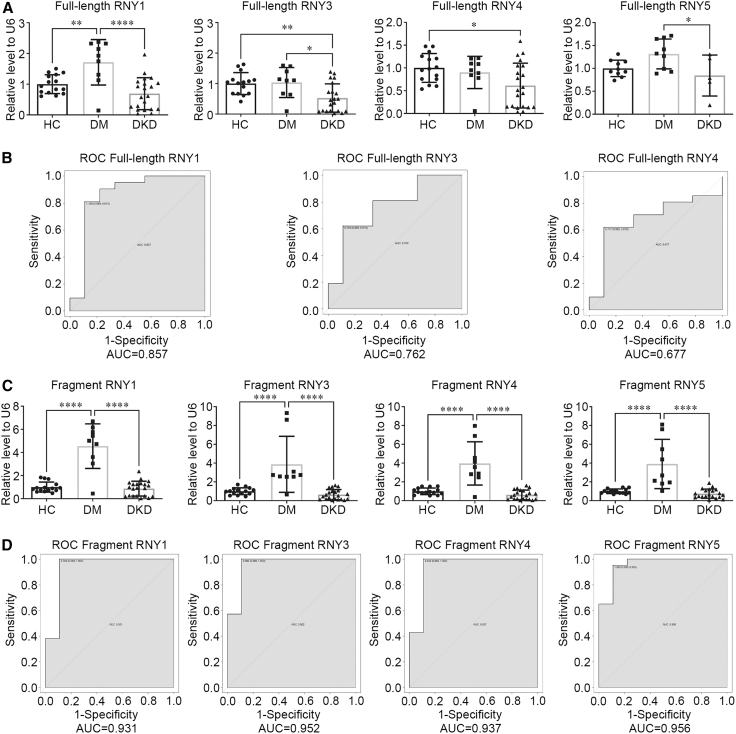
Differential expression and diagnostic performance of full-length and fragment RNYs in serum (A) The expression levels of full-length RNYs in serum differ among groups. (B) ROC curve for full-length RNYs to discriminate DKD patients from DM patients. (C) The expression levels of all fragment RNYs in serum differ among groups. (D) ROC curves of fragment RNYs showed good discriminative performance. Data are presented as mean ± SD. Statistical analysis was performed using one-way ANOVA followed by Tukey’s post hoc test. ROC analysis was used to assess diagnostic performance. Significance indicated by ∗*p* < 0.05, ∗∗*p* < 0.01, ∗∗∗∗*p* < 0.001.

We next sought to investigate whether circulating RNY levels associated with DM and DKD. First, we determined FL RNYs (Figure 1A), and observed that serum expression of RNY1, RNY3, and RNY5 in DKD patients was significantly lower than that in DM. Moreover, RNY1 exhibited significantly higher expression levels in the circulation of DM patients compared to both healthy control (HC) and DKD groups. RNY3 expression was significantly reduced in the DKD group relative to both the HC and DM groups. RNY4 levels were significantly lower in DKD patients than in the HC group, although no statistically significant difference was observed between the DKD and DM groups. The receiver operating characteristic (ROC) curve was employed to evaluate the ability of RNYs to distinguish DKD patients among individuals with DM (Figure 1B). The ROC curves of FL RNY1 and RNY3 demonstrated strong discriminatory performance. The area under the ROC curve (AUC) for RNY1 was 0.857, with a cutoff value of 1.108. Similarly, the AUC for FL RNY3 was 0.762, with a cutoff value of 0.569. In contrast, FL RNY4 exhibited poorer discriminative ability, yielding an AUC of 0.677. Important to note, although RNY5 significantly decreased in the DKD group compared to the DM group, the expression level was generally low, with most DKD patients exhibiting cycle threshold (Ct) values exceeding 40 or testing negative.

**Figure 2:**
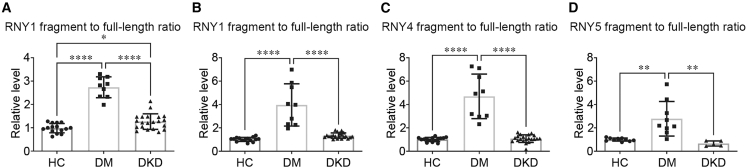
Altered fragment-to-full-length RNY ratios among HC, DM, and DKD groups (A–D) Fragment RNYs to full length ratios in serum across HC, DM, and DKD groups. Data are presented as mean ± SD. Statistical analysis was performed using one-way ANOVA followed by Tukey’s post hoc test. Significance indicated by ∗*p* < 0.05, ∗∗*p* < 0.01, ∗∗∗∗*p* < 0.001.

**Figure 3:**
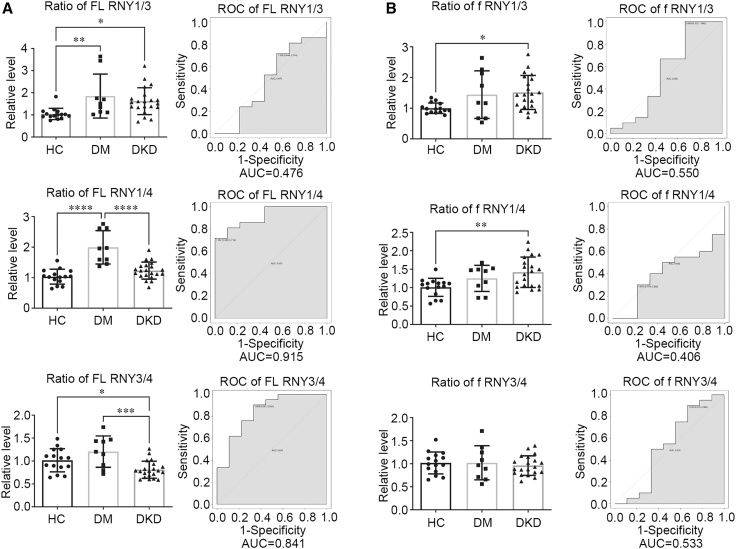
Diagnostic performance of full-length and fragment RNY subtype ratios in serum (A) Subtype ratios of specific full length RNYs in serum. ROC curves showed the possibility of discrimination DKD from DM patients. (B) Subtype ratios of specific fragment RNYs in serum. ROC curves indicated that all ratios of fragment RNYs are poor as biomarkers for kidney injury. Data are presented as mean ± SD. Statistical analysis was performed using one-way ANOVA followed by Tukey’s post hoc test. ROC analysis was used to assess diagnostic performance. Significance indicated by ∗*p* < 0.05, ∗∗*p* < 0.01, ∗∗∗∗*p* < 0.001.

For the full-length and fragmented Y-RNAs presented in Figures 1, 2, and 3, Ct values were generally within the following ranges: FL RNY1, Ct 27–31; FL RNY3, Ct 26–30; FL RNY4, Ct 25–29; FL RNY5, Ct 29–32; fRNY1, Ct 30–35; fRNY3, Ct 28–33; fRNY4, Ct 29–33; and fRNY5, Ct 31–35.

Next, we determined circulating RNY fragment expression levels. To validate the specificity of the quantitative reverse-transcription PCR (RT-qPCR) assays used to detect RNY fragments, endpoint PCR products generated using the same primer sets were analyzed by agarose gel electrophoresis, confirming the presence of single amplicons of the expected size (Figure S2). Interestingly, all the f RNYs in the DM group were significantly higher than those in the HC group, while the f RNYs in the DKD group were significantly lower than those in the DM group (Figure 1C). All f RNYs expression levels showed outstanding discrimination ability (Figure 1D): the AUC of f RNY1, RNY3, RNY4, and RNY5 are 0.931, 0.952, 0.937, and 0.956, respectively.

### Altered full-length to fragmented RNY ratios upon DM and DKD

Given that FL RNYs are precursors of f RNYs, the ratio of fragment to full length RNYs might reflect altered RNY processing and may hold biomarker potential or reflect underlying biological mechanisms. To that end we assessed these ratios, and we observed strongly increased ratios of fragment to full length RNYs upon DM as compared to HC, while these ratios decrease again upon DKD (Figure 2), suggesting DM and/or DKD specific effects.

### Altered RNY subtype ratios upon DM and DKD

Since it has been previously shown that unique quantitative ratios of (full-length) RNY subtypes may reflect cellular origin and different types of injury,^31^ we next assessed circulating RNY subtype ratios, both for full-length (Figure 3A) and fragment (Figure 3B) RNYs. RNY5 levels were excluded from these analyses given their low abundance. The ratio of FL RNY1 to RNY3 was elevated in the DM and DKD groups relative to the HC group. However, the ratio of fragmental RNY1 to RNY3 was elevated in the DKD group relative to the HC group, while the DM group only showed a trend of increase but was not statistically significant. The ROC analyses revealed that the ratios of FL RNY1 to RNY3 and f RNY1 to RNY3 lacked diagnostic significance in distinguishing diabetic patients with renal complications. The ratio of full length RNY1 to RNY4 in DM relative to HC was elevated, while the ratio in DKD relative to DM was decreased. The ratio of f RNY1 to RNY4 was only significantly elevated in DKD relative to HC. The ratio of FL RNY3 to RNY4 was significantly decreased in DKD relative to both HC and DM, while there was no difference in their fragment ratio. The ratios of FL RNY1 to RNY4 and RNY3 to RNY4 illustrated substantial discriminative ability, with AUC values of 0.915 and 0.841, respectively, indicating their potential utility in identifying kidney injury.

### The expression level of RNYs in different circulating carriers

**Figure 4:**
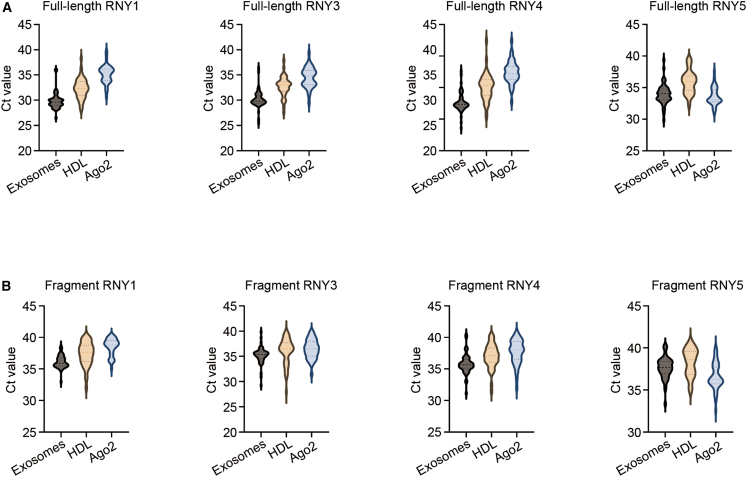
Distribution of full-length and fragment RNYs across circulating carrier fractions (A) Distribution of Ct values for full length RNYs in exosomes, HDL and Ago2 respectively. (B) Distribution of Ct values for fragment RNYs in exosomes, HDL and Ago2, respectively. Data are presented as distributions of Ct values across different carrier fractions.

RNYs, as a class of ncRNAs, are not stably present in the extracellular environment and require protective carriers for their stability and transportation. The currently identified ncRNAs carriers include EVs, high-density lipoprotein (HDL), and Argonaute 2 (Ago2), the latter mainly for miRNAs. Here, we have isolated these carriers and quantified the expression levels of RNYs within them. Due to the inherent challenge of a consistent reference across carrier samples, we have focused on analyzing the Ct values to determine the relative expression levels of RNYs. RNY1, RNY3, and RNY4 were predominantly found in EVs and to a lower extent carried by HDL and Ago2, whereas RNY5 exhibited a distinct distribution, with its expression in Ago2 being comparable to, or even exceeding, that in EVs and HDL (Figure 4A). A similar distribution, yet with less pronounced differences was observed for the fragments of RNY1, RNY3, RNY4, and RNY5 (Figure 4B).

### RNYs are present in urine and their levels increase upon DKD

**Figure 5:**
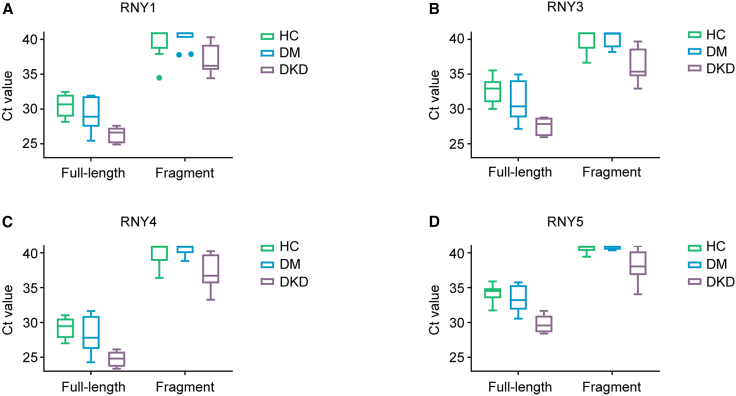
Detection patterns of full-length and fragment RNYs in urine across HC, DM, and DKD groups (A–D) Distribution of Ct values for full-length and fragment RNYs in urine across HC, DM, and DKD groups. Data are presented as distributions of Ct values.

Given that kidney injury may results in potential leakage of molecules into the urine, while urine assessment also provides a potential non-invasive alternative, we next assessed whether RNYs were detectable in the urine of patients and whether kidney injury affected their levels. Indeed, we observed that FL RNYs were detectable in urine with the FL RNYs exhibiting higher expression levels compared to their fragmented counterparts (Figure 5). In fact, f RNYs were consistently detectable only in the urine samples of patients with DKD, whereas they were less frequently detectable in the urines of either the DM patients or HCs. Interestingly, upon DKD we observed a strong increase in urinary RNY levels (Figure 5).

### RNYs correlate with clinical indicators of kidney and vascular injury

**Figure 6:**
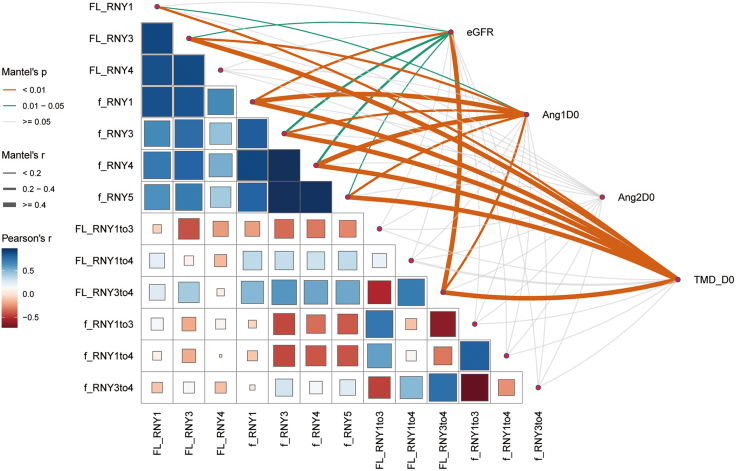
Correlation network between RNYs and clinical parameters in DKD Pearson’s correlation analysis was performed among full-length RNYs (FL_RNYs), RNY fragments (f_RNYs), and RNY subtype ratios. Mantel tests were used to assess associations between RNY-related variables and clinical indicators, including eGFR, Ang1D0, Ang2D0, and TMD_D0. Blue squares indicate positive Pearson’s correlations, whereas red squares indicate negative correlations, with color intensity corresponding to correlation strength. Connecting lines represent Mantel correlations between RNY-related variables and clinical parameters. Orange and green lines indicate statistically significant associations (*p* < 0.05), while gray lines represent non-significant associations. Line thickness corresponds to Mantel’s R value.

To investigate the potential clinical relevance of circulating RNYs, we assessed the correlation between RNY expression levels and key clinical indicators, including estimated glomerular filtration rate (eGFR). Given that DKD is strongly linked to vascular injury,^32^ and EV-linked RNY secretion has been linked to endothelial cell injury, we also assessed the association with angiopoietin-1/2 (Ang1/Ang2) and soluble thrombomodulin (sTM) levels as markers of vascular injury. Using Mantel test correlation analysis, we identified significant associations between specific RNYs and these clinical parameters (Figure 6). Notably, no significant associations were observed for RNY4. In contrast, eGFR, Ang1, and sTM showed strong associations with FL RNY1, RNY3, the RNY3/RNY4 ratio, and multiple fragment-derived RNYs, suggesting a link to kidney and vascular injury.

## Discussion

RNYs have received relatively little attention as compared to traditional ncRNA such as miRNAs and lncRNAs, and mainly focused on the fields of cancer and inflammation-related diseases.^16^^,^^23^^,^^27^^,^^33^ Here, we studied circulating RNYs in DKD, which has not been investigated before, to identify potential novel and effective biomarkers for kidney damage which may improve diagnosis and ultimately treatment of DKD.

We found that FL RNY1, RNY3, and RNY5 in the serum were significantly reduced in the DKD group compared to the DM group, with good performance in ROC curves, suggesting that RNYs could potentially serve as biomarkers for diabetic kidney injury. The levels of fragmented RNYs in the serum showed a similar trend, with significantly lower expression in the DKD group than in the DM group. Interestingly, all fragmented RNYs were found at higher levels in the DM group than in the HC group, indicating that the aberrant physiological processes in diabetes may be associated with fragmentation of RNYs, warranting further investigation. In addition, we calculated the ratio of the expression levels of each fragmented RNY to its full-length counterpart, a ratio that may reflect the amount of fragmentation of RNYs. All ratios of fragmented RNYs to FL RNYs showed the same trend, with a significant increase in the DM group and a significant decrease in the DKD group. Given that the expression of FL RNYs did not change between the DM and HC groups (except for an increase in RNY1 in the DM group), it indicates that the differences in expression between fragmented and FL RNYs in the DM and HC groups stem from increased amounts of fragmented RNYs, suggesting that RNYs undergo more splicing in the diabetic state. The decrease of this ratio upon DKD is yet more complex. This may either reflect less fragmentation, or increased excretion from the body through the damaged kidneys. Therefore, we tested the levels of RNYs in the urine of the three groups of patients and consistently found substantial levels of fragmented RNYs in the urine of the DKD group, while in the other groups the RNY fragments were hardly detectable. This suggests that fragmented RNYs in urine may reflect kidney damage-related alterations in RNY processing.

ncRNA molecules are highly unstable in the extracellular environment and are easily degraded by RNases, thus requiring carriers for protection to exist stably. Several known RNA carriers include EVs, HDL, and Ago2.^34^ RNYs, as a type of ncRNA, may also be protected by these carriers. Consequently, we extracted these three components from circulating blood and measured their RNYs expression levels. Our research indicates that RNY1, RNY3, and RNY4 are predominantly found in EVs (in line with previous findings)^18^ and HDL, yet also detectable in Ago2, suggesting potential miRNA-like functions.^29^^,^^34^^,^^35^ RNY5 exhibits a different distribution, with its expression in Ago2 being comparable to, or even higher than, that in EVs and HDL. ncRNAs carried by different carriers may perform distinct functions,^35^ and it is worth investigating whether RNY5 differs in function from other RNYs.

It has been previously demonstrated that the ratio of different RNY subtypes in exosomes can indicate inflammatory states, and EVs released from different blood cells have varying Y-RNA subtype ratios.^31^ For instance, neutrophil EVs have a high Y4/Y3 ratio, whereas peripheral blood mononuclear cells (PBMCs) have a high Y3/Y1 ratio. Interestingly, we found that compared to the DM group, the ratios of FL RNY1/4 and RNY3/4 in the serum were significantly reduced in the DKD group, while the ratio of RNY1/3 increased upon DM and DKD. These findings thus may reflect pro-inflammatory conditions, and increased neutrophil and PBMC activity, respectively. However, further studies are required to pinpoint this exact relation.

We previously found DKD to associate with increased markers of vascular injury.^32^ In addition, a remarkable enrichment was previously observed of RNYs released in EVs by endothelial cells,^36^ suggesting a relation between RNYs and vascular function. As such, we determined correlations with vascular injury markers and found that RNY1, 3, and 5 and all fragments RNYs were strongly correlated with vascular injury markers, in particular sTM. These findings suggest RNYs may be functionally related to kidney and vascular pathophysiology.

Interestingly, our correlation analysis (Mantel test) revealed that clinical indicators were associated with RNY3 rather than RNY4. Given the close genomic proximity of RNY3 and RNY4, it is worth investigating whether its transcriptional regulation plays a role in diabetic kidney injury.

Despite these findings, several limitations should be acknowledged. First, normalization using an exogenous spike-in control was not performed, as samples were processed retrospectively without the inclusion of synthetic controls such as cel-miR-39 during RNA isolation. This may affect the accuracy of comparisons across samples and carrier fractions. In addition, the lack of a universally accepted endogenous reference for different plasma carrier fractions further complicates normalization. Therefore, Ct values were primarily used to assess relative trends and detectability of RNYs rather than absolute expression levels. Furthermore, the analysis of urine samples may be influenced by variations in sample concentration, which could also impact Ct-based comparisons. Nonetheless, our small ncRNA-based normalization suggests similar outcomes as with reported Ct values. Yet, validation in blinded and independent sample cohorts will be important to further assess the reproducibility and clinical applicability of the current RT-qPCR-based approach.

It was previously demonstrated that RNA-seq studies both detected fragments derived from both the 5′ and 3′ arms,^37^ which is also supported by our own exploratory RNA-seq analysis, whereas other studies mainly reported full-length Y-RNAs.^38^^,^^39^ Thus, from the literature a picture emerges that, in contrast to cells that mainly appear to harbor full length yRNAs, circulating yRNAs mainly consist of fragments. However, another study suggested that the secondary structure of RNYs might impact full-length cDNA synthesis, resulting in underestimation of full length yRNAs in sequencing data.^20^ Further studies should be performed to clarify this issue.

In summary, we find RNYs and their fragments to strongly associate with diabetes and DKD. Our findings indicate that RNYs deserve attention as possible biomarkers for kidney injury in DKD. Their relation with inflammation and vascular injury warrant further research to elucidate the precise mechanisms by which RNYs function in DKD. This ultimately could lead to the development of new therapeutic strategies to control disease progression and improve patient outcomes.

## Materials and methods

### Clinical samples

This study was approved by the institutional review board (Leiden University Medical Center, Leiden, the Netherlands) and complied with the ethical principles of the Declaration of Helsinki. Informed consent was obtained from all studied patients. The main study cohort comprised a control group (*n* = 15) of healthy, age-matched volunteers; a group of patients with type 1 DM (*n* = 9); and a group of patients with type 1 DM with DKD with an eGFR <30 mL/min/1.73 m2 (*n* = 21). Table 1 shows the basic information of the patients.

**Table 1:** Baseline characteristics of the study population (*n* = 45)

	HC (*n* = 15)	DM (*n* = 9)	DKD (*n* = 21)
Gender. male (%)	7 (46.7)	4 (44.4)	16 (76.2)
Age (years)	45.6 ± 11.49	50 ± 14.13	43.95 ± 5.47
BMI (kg/m^2^)	25.65 ± 4.11	23.22 ± 1.75	25.39 ± 3.19
Systolic blood pressure (mmHg)	132.67 ± 12.71	129.22 ± 16.56	145.52 ± 19.28
Diastolic blood pressure (mmHg)	82.33 ± 7.24	70 ± 6.61	86.24 ± 10.81
Hemoglobin (mmol/L)	8.6 ± 0.63	8.87 ± 1.1	7.55 ± 0.52
Hematocrit (L/L)	0.41 ± 0.02	0.42 ± 0.04	0.36 ± 0.03
HbA1c (%)	ND	7.29 ± 0.63	8.94 ± 2.29
Glucose (mmol/L)	5.21 ± 0.97	12.2 ± 6.06	13.83 ± 6.39
eGFR (mL/min/1.73 m2)	96.6 ± 14.92	86.23 ± 8.65	18.35 ± 7.47
Proteinuria (g/24 h)	ND	ND	1.36 ± 1.58

N/D, not determined.

### Clinical samples and data collection

Blood samples were procured from a peripheral arm vein, with subsequent collection into tubes containing a clotting activator for serum separation and tubes coated with ethylenediaminetetraacetic acid (EDTA) for plasma separation. The serum collection tubes were gently inverted 3–6 times to ensure thorough mixing and then allowed to coagulate for a period of 30 min at ambient temperature prior to centrifugation at 2,000 × g for 10 min. Plasma was separated from whole blood by centrifugation of EDTA-anticoagulated samples at 1,000 g for 10 min. Depending on their volume the supernatants were transferred into 3–4 smaller tubes in 200–600 μL aliquots and immediately frozen at −80°C until further processing. Plasma creatinine, hemoglobin, HbA1c, glucose, and urea were measured as well as proteinuria in 24-h urine. GFR was calculated with plasma creatinine concentration using the MDRD equation. Urine sample was collected and centrifuged at 4,700 × *g* for 15 min at 4°C. The supernatant was then aliquoted and stored at −80°C for following analysis.

### EVs, HDL, and Ago2 particles isolation

For the EV, HDL, and Ago2 analyses, archival material was used and isolations were performed as previously described.^34^ In short, 125 μL human plasma was applied to a 3.64-mL Sepharose CL-2B size exclusion chromatography (SEC) column. Different fractions were analyzed for the presence of particles using nanoparticle tracking analysis (NS500; NanoSight, Amesbury, UK). The presence of EVs was confirmed by western blot and transmission electron microscopy (TEM). EV eluate was applied to an Amicon Ultra-4 Centrifugal Filter Unit for concentration and treated with proteinase K (Thermo Fisher Scientific, Landsmeer, the Netherlands).

HDL (density 1.063–1.21 g/mL) was isolated with potassium bromide (KBr) density gradient ultracentrifugation (DGUC) using an Optima Max benchtop ultracentrifuge with a fixed-angle TLA 110 rotor. Plasma aliquots of 900 μL were adjusted to the desired density with solid KBr and ultracentrifuged in 13 × 48 mm polycarbonate tubes at 100,000 rpm (435,680 g) for 2 h at 10°C. Apolipoprotein A1 (apoA1) concentration was analyzed using an immunoturbidimetric assay. The HDL fraction was isolated, dialyzed for 2 h against PBS for KBr removal, applied to an Amicon Ultra-4 Centrifugal Filter Unit for further concentration, and applied to a sepharose-CL-2B SEC column to deprive HDL from contamination with CD63+ EVs.^29^

For ago-2, goat anti-mouse IgG magnetic beads (Thermo Fisher Scientific) were incubated with 10 μg mouse monoclonal anti-human Ago-2 (ab57113; Abcam) or mouse normal IgG (Santa Cruz Biotechnology) antibodies for 2 h at 4°C. Preincubated Ago-2 beads were added to 400 μL diluted plasma and incubated overnight at 4°C. Beads were washed three times with 1% Nonidet P-40 buffer (1% Nonidet P-40, 50 mmol/L Tris-HCl, pH 7.4, 150 mmol/L NaCl, 2 mmol/L EDTA) and split in half for RNA isolation and western blot analysis.

### RNA-seq data

The acquisition of circulating RNA-seq data samples was performed as described in previous studies.^40^ The human reference genome GRCh38.p14 was employed for alignment and subsequent analyses. Sequencing data were processed and analyzed using the gviz and GenomicRanges packages within the R programming environment. Manual alignment of RNY loci was performed as follows: BAM files generated by the exceRpt pipeline were processed in R using the packages Rsamtools (v.3.23) and GenomicRanges (v.3.23). Genomic loci of interest were imported from an Excel file and converted into GRanges objects. BAM files were subsequently sorted and indexed using the sortBam() and indexBam() functions. Reads overlapping the specified genomic regions were quantified with countBam() using region-specific ScanBamParam settings. Read counts were aggregated per locus and sample into a single table for downstream analyses and visualization. Data visualization was performed using ggplot2 (v.4.0.3).

### RNA isolation and quantitative RT-PCR

RNA from Serum, EVs, HDL, and Ago-2 fractions was isolated with 800 μL TRIzol reagent (Invitrogen, Breda, the Netherlands) using the RNeasy Micro Kit (QIAGEN, Venlo, the Netherlands). MiScript RT II kit (QIAGEN, Hilden, Germany) was used for cDNA making. Equal input volumes of RNA (3 μL per reaction) were used with HiFlex buffer, nucleics mix and reverse transcriptase mix in kit. The incubation program was set as 60 min in 37°C and 5 min in 95°C as the instruction. Then the SYBR Select Master Mix (Applied Biosystems 4472908) was used for subsequent RT-qPCR. Reverse primers for f RNYs (Universal Primers) and human U6 primers (RNU6-5p) were provided by MiScript RT II kit. Other primers were shown in Table 2. Specificity of the primers were checked through using the melting curve data and ct values of 0 and >39 were removed to ensure reliability. Relative expression levels were normalized against U6 snRNA as the endogenous reference control and calculated using the 2ˆ-ΔCt method.

**Table 2:** Primers

Gene	Sequence
Fragment RNY1	GATCGAACTCCTTGTTCTACTC
Fragment RNY3	AGATTTCTTTGTTCCTTCTCCACTC
Fragment RNY4	GTGTCACTAAAGTTGGTATACAAC
Fragment RNY5	GTTAAGTTGATTTAACATTGTCTC
hsaRNY1 F	GGCTGGTCCGAAGGTAGTGAG
hsaRNY1 R	GGGGGAAAGAGTAGAACAAGG
hsaRNY3 F	CCGAGTGCAGTGGTGTTTAC
hsaRNY3 R	AAGCAGTGGGAGTGGAGAA
hsaRNY4 F	TCCGATGGTAGTGGGTTATCA
hsaRNY4 R	AAAGCCAGTCAAATTTAGCAGT
hsaRNY5 F	AGTTGGTCCGAGTGTTGTGG
hsaRNY5 R	AACAGCAAGCTAGTCAAGCG
mmuRNY1 F	GGCTGGTCCGAAGGTAGTGAG
mmuRNY1 R	GGGGGGAAAGTGTAGAACAGG
mmuRNY3 F	GGTCCGAGAGTAGTGGTGTTTAC
mmuRNY3 R	AAGCAGTGGGAGCGGAGAA

### Statistical analyses

Continuous, normally distributed data are presented as mean ± SD. Variable distribution was tested using the Kolmogorov-Smirnov test for normal distribution. Differences between groups in the main study were analyzed using one-way ANOVA. Categorical variables are presented as numbers and percentages, and differences between two groups were analyzed with Fisher exact test. Differences were considered statistically significant with *p* < 0.05. As this study was exploratory in nature, no formal sample size calculation was performed prior to patient inclusion. An additional sample size estimation was performed using the Select Statistical Services sample size calculator for comparing two means, based on the observed difference (1.02) and variance (0.74). The analysis indicated that approximately 12 subjects per group would be required to achieve 80% power at a 95% confidence level. Data analysis was performed using SPSS v.25 (IBM Corporation, Chicago, IL, USA) and GraphPad Prism v.7.0 (GraphPad Software, San Diego, CA, USA) statistical software. Correlation and mantel test analysis were performed by linkET and vegan package in R.

### Ethics approval

This study was approved by the institutional review board (Leiden University Medical Center, Leiden, the Netherlands) and complied with the ethical principles of the Declaration of Helsinki. Informed consent was obtained from all studied patients.

## Data availability

The authors declare that the main data supporting the findings of this study are available within the article and its supplementary Information file.

## Acknowledgments

Q.Z. and Y.L. were supported by a scholarship from 10.13039/501100004543China Scholarship Council. R.B. was supported by a grant from the 10.13039/501100002997Dutch Kidney Foundation (20OK015) and EFSD/Novo Nordisk Foundation Future Leaders awards program (NNF23SA0087433).

## Author contributions

Conceptualization, R.B., Q.Z., Y.L., and A.J.v.Z.; methodology, Q.Z., Y.L., R.B., R.P., J.M.G.J.D., J.A.d.K., L.M.H., J.I.R., and A.J.v.Z.; investigation, Q.Z., Y.L., R.P., J.M.G.J.D., J.A.d.K., and R.B.; writing – original draft, R.B. and Q.Z.; writing – review and editing, R.B., Y.L., J.A.d.K., R.P., A.J.v.Z., and J.I.R.; funding acquisition, R.B., A.J.v.Z.; supervision, R.B., L.M.H., J.I.R., and A.J.v.Z.

## Declaration of interests

The authors declare no competing interests.

## Supplemental information

Document S1. Figures S1–S4

Document S2. Article plus supplemental information
