# Supplementary material for: Y-RNAs and their fragments reflect kidney injury in diabetic kidney disease
Source: Mol Ther Nucleic Acids. 2026 Jul 1;37(3):102998. doi: 10.1016/j.omtn.2026.102998 (PMC13382184; doi:10.1016/j.omtn.2026.102998)
Supplement: Document S1. Figures S1–S4 [file mmc1.pdf]

OMTN, Volume *37*

Supplemental information

Y-RNAs and their fragments reflect kidney injury in diabetic kidney disease

Qiao Zhao, Yunyi Liang, Rudmer Postma, Jacques M.G.J. Duijs, Juliette A. de Klerk, Leen M. 't Hart, Joris I. Rotmans, Anton Jan van Zonneveld, and Roel Bijkerk

A

RNY1 (112 nt)

GGCTGGTCCGAAGGTAGTGAGTTATCTCAATTGATTGTTCACAGTCAGTTACAGATCGAACTCCTTGTTC
TACTCTTTCCCCCCTTCTCACTACTGCACTTGACTAGTCTTTT

RNY3 (101 nt)

GGCTGGTCCGAGTGCAGTGGTGTTTACAACTAATTGATCACAACCAGTTACAGATTTCTTTGTTCCTTCTCCACTCCCACTGCTTCACTTGACTAGCCTTTT

RNY4 (93 nt)

GGCTGGTCCGATGGTAGTGGGTTATCAGAACTTATTAACATTAGTGTCACTAAAGTTGGTATACAACCCCCCACTGCTAAATTTGACTGGCTTTTT

RNY5 (83 nt)

AGTTGGTCCGAGTGTTGTGGGTTATTGTTAAGTTGATTTAACATTGTCTCCCCCCACAACCGCGCTTGACTAGCTTGCTGTTTT

B

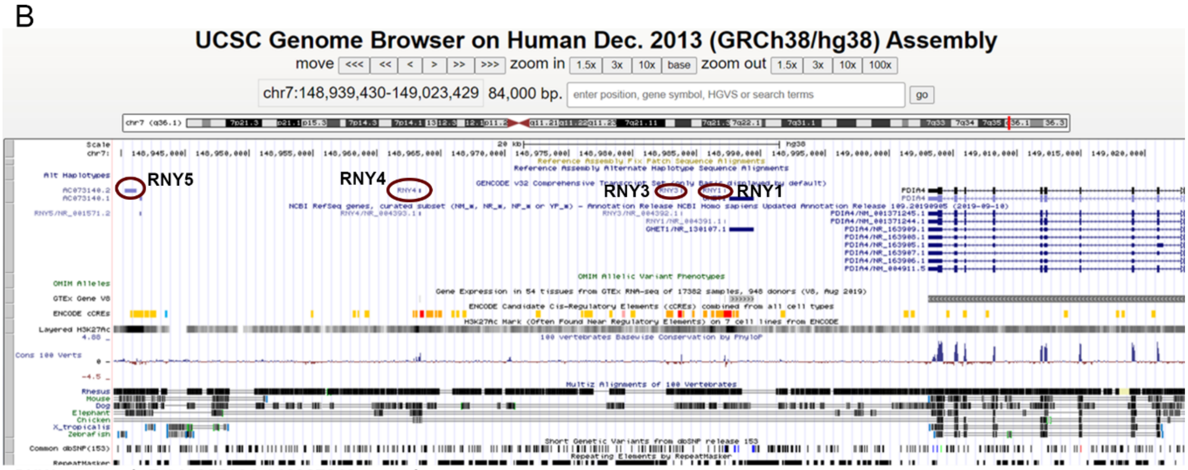

RNY1: range=chr7:148987136-148987248 strand= -

RNY3: range=chr7:148983755-148983856 strand=+

RNY4: range=chr7:148963315-148963410 strand=+

RNY5: range=chr7:148941488-148941571 strand=+

C

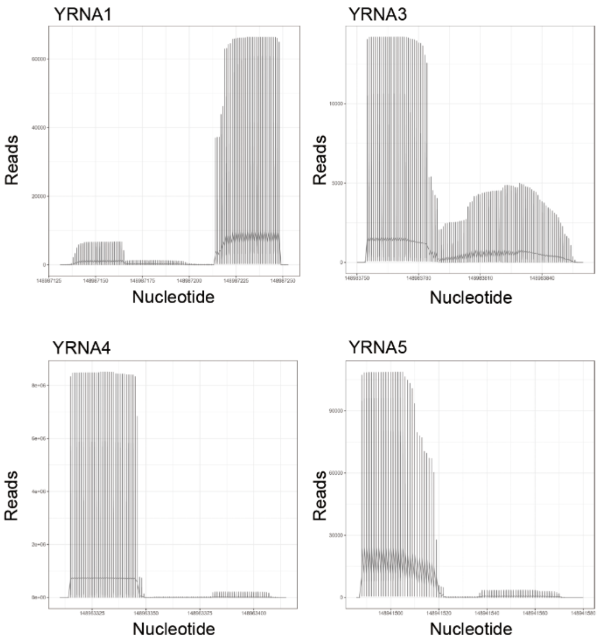

D

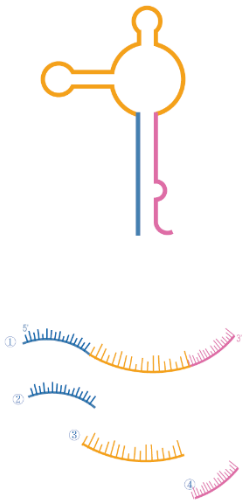

E

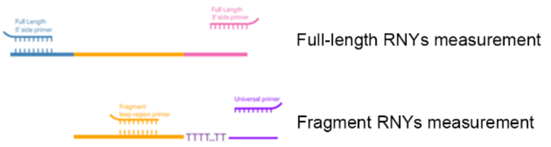

Figure S1

Figure S1. Full-length YRNA sequences, genomic location, RNA-seq coverage, YRNA structure, and primer positioning.

(A) Full-length sequences of RNY1, RNY3, RNY4, and RNY5. (B) Genomic location of YRNA genes on chromosome 7 based on the UCSC Genome Browser. (C) RNA-seq read coverage across the genomic loci of RNY1, RNY3, RNY4, and RNY5. Note that the direction of RNY1 is from right to left. (D) Schematic diagram of the secondary structure of RNYs and full-length and fragmented forms of YRNAs. The ① full-length yRNA can be cleaved into a ② 5'-terminal fragment, a ③ middle loop region fragment, and a ④ 3'-terminal fragment. (E) Schematic representation of primer positioning for full-length and fragmented YRNA detection.

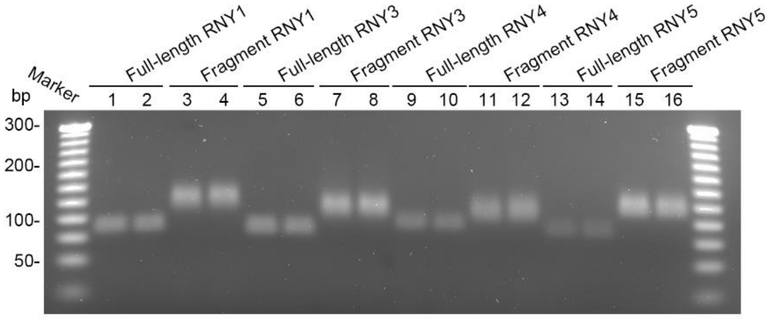

Figure S2

Figure S2. Electrophoretic validation of full-length and fragment YRNAs using distinct primers.

Endpoint PCR products corresponding to full-length YRNA (RNY1, RNY3, RNY4, and RNY5) and YRNA fragments were analyzed by agarose gel electrophoresis. Predominant bands were detected in the expected size ranges. Fragment products showed a higher apparent molecular weight than full-length amplicons, consistent with the poly(A)-tailing–based amplification approach. DNA size markers (bp) are shown.

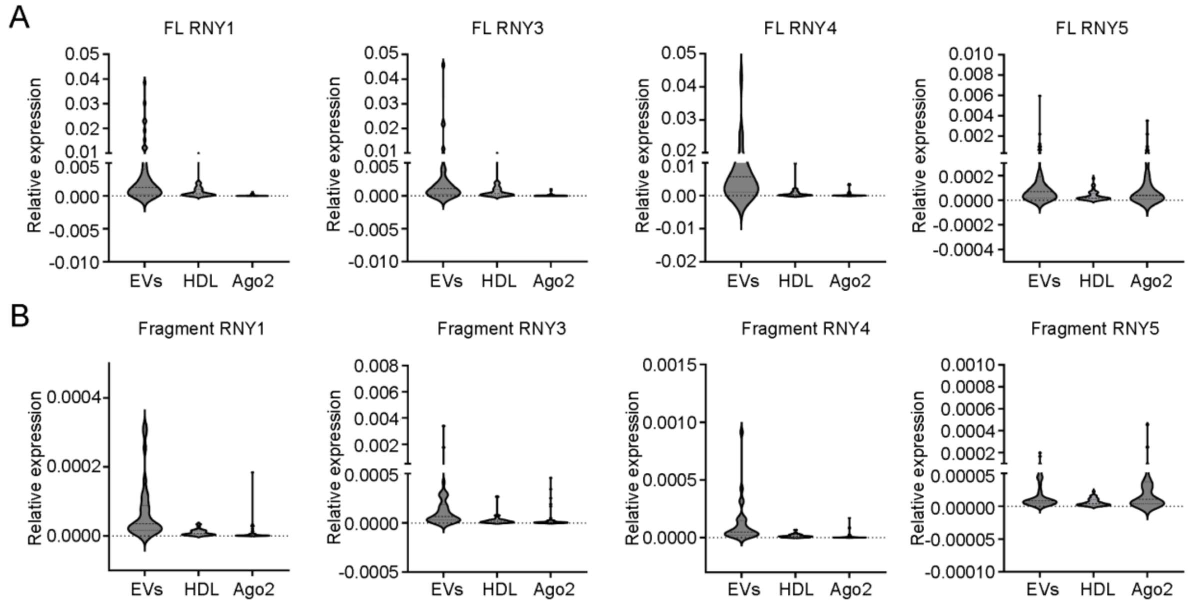

Figure S3

Figure S3. Additional normalization analyses of RNY expression across carrier fractions.

(A) Relative expression levels of full-length (FL) RNY1, RNY3, RNY4, and RNY5 across extracellular vesicles (EVs), high-density lipoprotein (HDL), and Argonaute 2 (Ago2) carrier fractions following normalization to miR16. (B) Relative expression levels of corresponding fragmented RNY species across EVs, HDL, and Ago2 fractions after miR16 normalization.

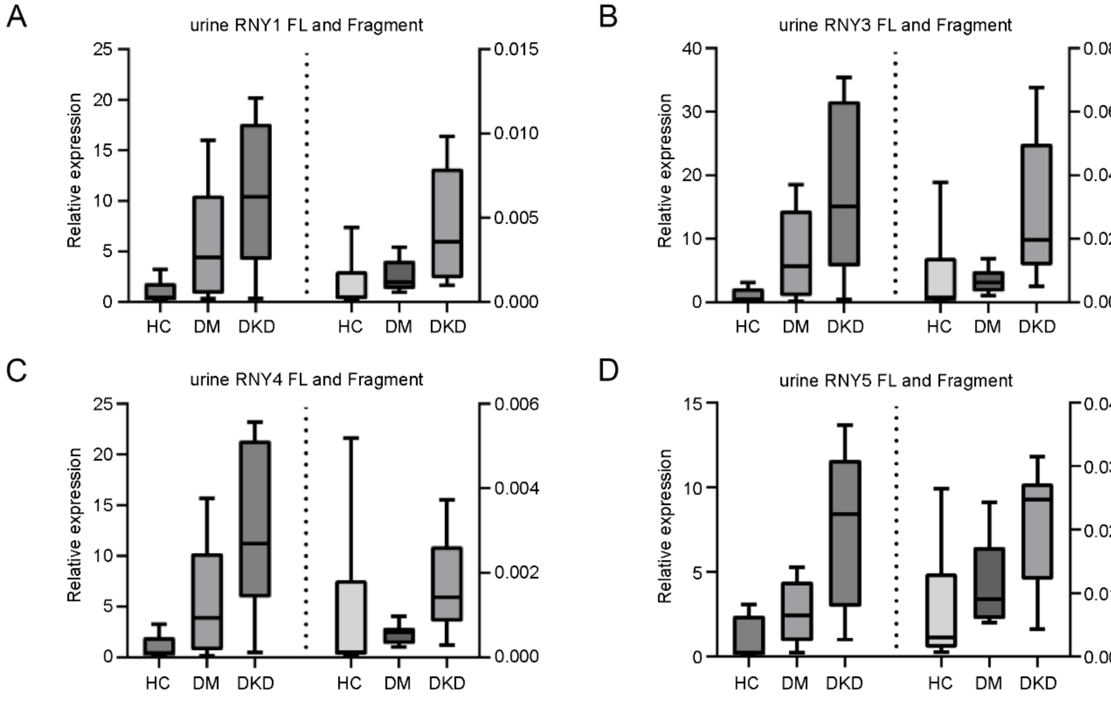

Figure S4

Figure S4. Additional normalization analyses of RNY expression across carrier fractions.

(A–D) Relative expression levels of full-length and fragmented RNY1 (A), RNY3 (B), RNY4 (C), and RNY5 (D) in urine samples following normalization to U6. Data are shown for healthy controls (HC), diabetes mellitus (DM), and diabetic kidney disease (DKD). For each panel, full-length and fragment RNY expression levels are presented on separate axes (left and right, respectively).
